# Fluorescence Quenching Effects of Carbon Shells on Eu_2_O_3_ Nanoparticles

**DOI:** 10.3390/ijms27146436

**Published:** 2026-07-20

**Authors:** Abdullah Khamis Ali Al Saidi, Huan Yue, Tirusew Tegafaw, Dejun Zhao, Ying Liu, Endale Mulugeta, Xiaoran Chen, Ziyi Lin, Ahrum Baek, Jihyun Kim, Jae Chang Jung, Hunkyu Ryeom, Yongmin Chang, Gang Ho Lee

**Affiliations:** 1 Department of Chemistry, College of Natural Sciences, Kyungpook National University, Daegu 41566, Republic of Korea; abdullahalsaidi0429@gmail.com (A.K.A.A.S.); yuehuan66@outlook.com (H.Y.); tirukorea@gmail.com (T.T.); djzhao.chem@gmail.com (D.Z.); ly1124161@gmail.com (Y.L.); endexindex05@gmail.com (E.M.); tsukiyovo@gmail.com (X.C.); 01linziyi@gmail.com (Z.L.); 2 Institute of Biomedical Engineering Research, School of Medicine, Kyungpook National University, Daegu 41944, Republic of Korea; baxun@naver.com; 3 Department of Chemistry Education, Teachers’ College, Kyungpook National University, Daegu 41566, Republic of Korea; jkim23@knu.ac.kr; 4 Department of Biology, College of Natural Sciences, Kyungpook National University, Daegu 41566, Republic of Korea; jcjung@knu.ac.kr; 5 Department of Radiology, School of Medicine, Kyungpook National University, Daegu 41944, Republic of Korea; hkryeom@knu.ac.kr; 6 Department of Molecular Medicine, School of Medicine, Kyungpook National University, Daegu 41944, Republic of Korea

**Keywords:** carbon shell, fluorescence quenching, Eu_2_O_3_ nanoparticle, photoluminescence, quantum yield

## Abstract

The performance of fluorescent nanoparticles is significantly affected by fluorescence quenching materials, such as impurities. In this study, the fluorescence quenching effects of carbon shells on Eu_2_O_3_ nanoparticles were investigated by synthesizing bare and carbon-grafted Eu_2_O_3_ nanoparticles with different amounts of grafted carbon using dextrose as a carbon source to simulate carbon contaminations and investigate this issue and by measuring their photoluminescence (PL) spectra and absolute quantum yields (QYs). The synthesized Eu_2_O_3_@C core-shell nanoparticles were further grafted with the organic photosensitizer 2,6-pyridinedicarboxylic acid (PDA) to enhance their QYs. It was found that as the grafted carbon amount increased, the PL intensity and QY decreased, demonstrating the fluorescence quenching effect of the grafted carbon layers. Therefore, it is better to avoid carbon contaminations in fluorescent nanoparticles during synthesis in order to achieve high QYs.

## 1. Introduction

Fluorescence imaging (FI) plays a significant role in advancing biomedical sciences in the area of cellular and biological processes, disease diagnosis, and drug development [1,2,3]. To realize high-performance FI, fluorescent materials should possess high quantum yields (QYs). However, their QY can be reduced by contamination with fluorescent quenching materials. Therefore, fluorescent materials should be carefully synthesized under controlled conditions, avoiding contamination by quenching materials.

Recently, nanoparticles have emerged as a groundbreaking solution in FI applications owing to their excellent optical and physicochemical properties [2,4,5,6]. They do not undergo photobleaching and photodissociation as solid-state materials. They can exhibit remarkably stable fluorescence under conditions involving pH fluctuations, temperature changes, and ionic strength variations via surface modifications [7]. Another advantage is represented by their tunable optical properties and surface chemistry enabling conjugation with drugs and specific ligands that can target distinct biological markers, providing enhanced sensitivity in biomedical imaging [8]. Among fluorescent nanoparticles, lanthanide oxide nanoparticles are promising potential candidates owing to their unique optical properties such as large Stokes shifts (>200 nm), atomic-like sharp peaks, and long emission lifetimes, making them unaffected by interference from excitation wavelengths, distinguishable from broad background signals, and free from short-lived background signals via time-resolved fluorescence, respectively [9,10,11,12].

Fluorescence quenching, denoting the process that reduces the fluorescence intensity and QY of a material, significantly limits bioimaging performance. This process can be caused by various factors such as molecular interactions, molecular rearrangements, energy transfer, complex formation, and collisions [13,14,15,16,17]. Therefore, it is crucial to avoid contamination by quenching materials. It is well-known that carbon materials such as carbon dots and carbon sheets absorb and emit photons in the visible region [18,19,20] and may cause fluorescence quenching. However, fluorescence quenching by carbon has not been investigated in previous studies. Nanoparticles can be easily contaminated by carbon materials during synthesis, especially in high-temperature synthesis, because solvents and surface-grafting ligands mostly consist of organic molecules; hence, it is highly valuable to investigate the fluorescence quenching effects of carbon materials in order to design highly fluorescent nanoparticles.

In this study, we systematically investigated the carbon effects on the photoluminescence (PL) spectra and absolute QYs of europium oxide (Eu_2_O_3_) nanoparticles by synthesizing bare and carbon-grafted Eu_2_O_3_ nanoparticles with different amounts of grafted carbon layers to simulate carbon contaminations and investigate this issue using dextrose as a carbon source. The Eu_2_O_3_@C core-shell nanoparticles were stable in colloidal form in aqueous media owing to the presence of numerous -OH groups (originating from dextrose) on the outer carbon layer surfaces [21,22]. In addition, the Eu_2_O_3_@C nanoparticles were further grafted with the organic photosensitizer 2,6-pyridinedicarboxylic acid (PDA) to enhance their QYs. The PL spectra and absolute QYs of the PDA-Eu_2_O_3_@C nanoparticles with varying grafted carbon contents were measured in aqueous media. To further confirm the fluorescent quenching effect of carbon, the grafted carbon layers were removed by oxidation with hydrogen peroxide (H_2_O_2_) under 365 nm ultraviolet (UV) irradiation, followed by evaluating the corresponding PL intensity enhancements.

## 2. Results

### 2.1. Physicochemical Properties

#### 2.1.1. Particle Diameter, Hydrodynamic Diameter, Colloidal Stability, and Crystallinity of PDA-Eu_2_O_3_@C (Core = Eu_2_O_3_; Shell = Carbon Layer) Nanoparticles

**Figure 1:**
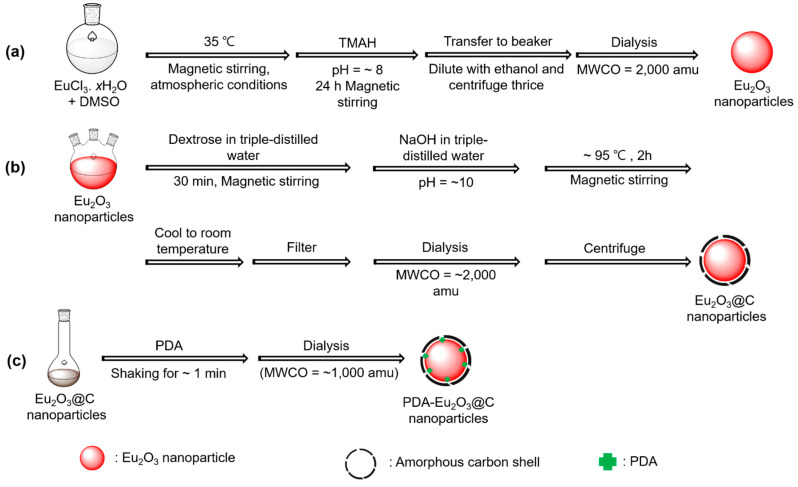
Synthesis schemes of (**a**) Eu_2_O_3_ nanoparticles, (**b**) Eu_2_O_3_@C core-shell nanoparticles (core: Eu_2_O_3_ and shell: carbon), and (**c**) PDA-Eu_2_O_3_@C nanoparticles.

**Figure 2:**
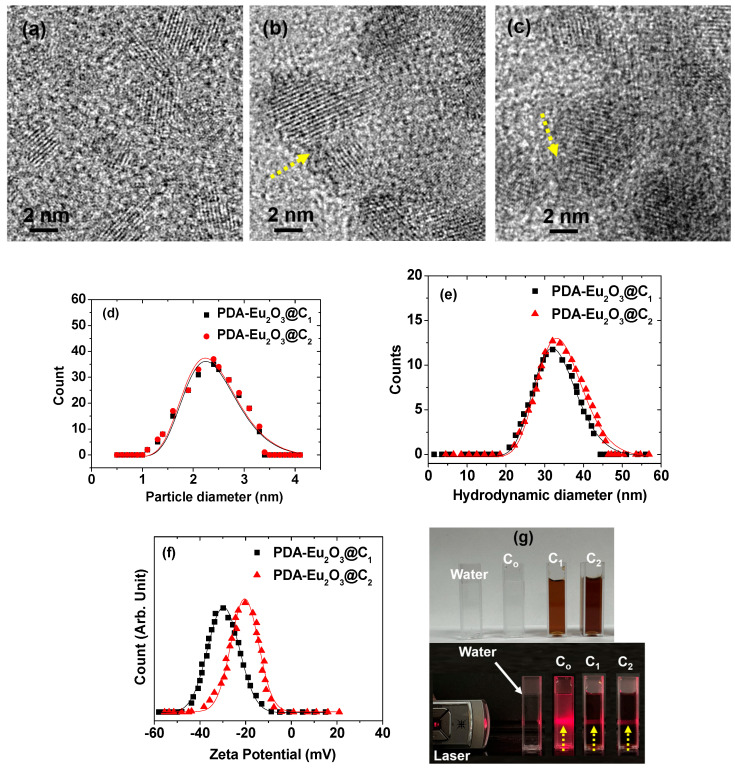
HRTEM images of PDA-Eu_2_O_3_@C [C = (**a**) C_0_, (**b**) C_1_, and (**c**) C_2_] nanoparticles; dotted arrows indicate grafted carbon layers. (**d**) Particle diameter distributions and log-normal function fits to obtain d_avg_ values. (**e**) Hydrodynamic diameter distributions and log-normal function fits to obtain a_avg_ values. (**f**) Zeta potential curves and Gaussian function fits to obtain ζ_avg_ values. (**g**) Photographs of aqueous suspension samples (~20 mM [Eu]), showing the absence of nanoparticle precipitation for >1.5 years after synthesis (top), as well as Tyndall effects (i.e., laser scattering, marked by dotted arrows), demonstrating colloidal dispersion of the nanoparticles, whereas no laser scattering was observed from a vial containing triple-distilled water (bottom).

Eu_2_O_3_ nanoparticles (labeled as Eu_2_O_3_@C_0_) were first synthesized (Figure 1a) and then grafted with different amounts of carbon by using varying amounts of dextrose as carbon precursor (with the obtained materials labeled as Eu_2_O_3_@C, C = C_1_ and C_2_; Figure 1b). The nanoparticles were further conjugated with the organic photosensitizer PDA (Figure 1c) to enhance their QYs. All synthesized PDA-Eu_2_O_3_@C nanoparticles exhibited ultrasmall particle size, as shown in the high-resolution transmission electron microscopy (HRTEM) images in Figure 2a–c. The HRTEM images also show the grafted carbon shells at the border of the Eu_2_O_3_ nanoparticle (as marked by dotted arrows in Figure 2b,c) with approximate carbon layer thicknesses of ~0.1 and ~0.2 nm for C = C_1_ and C_2_, respectively, whereas the Eu_2_O_3_ nanoparticles (C = C_0_) without grafted carbon layers exhibited no such carbon shells (Figure 2a). The distributions of the nanoparticle core (i.e., Eu_2_O_3_) diameters were fitted to a log-normal function, and the corresponding average diameter (d_avg_) was estimated to be 2.4 nm for both C = C_1_ and C_2_ (Figure 2d and Table 1). Moreover, hydrodynamic diameters were estimated from dynamic light scattering (DLS) patterns, which were fitted to a log-normal function to obtain the corresponding average hydrodynamic diameters (a_avg_; Figure 2e and Table 1). The synthesized PDA-Eu_2_O_3_@C nanoparticles exhibited highly negative zeta potentials (ζ_avg_) (Figure 2f and Table 1). They did not precipitate for more than 1 year after synthesis; as shown at the top of Figure 2g, the nanoparticle solution color had a dark-brown color after carbon grafting (C = C_1_) and became darker upon further carbon grafting (C = C_2_) owing to the black color of carbon nanomaterials. Additionally, the colloidal dispersion of the nanoparticles was demonstrated through visible light scattering, i.e., the Tyndall effect, whereas triple-distilled water (used as a reference) did not exhibit such scattering (bottom figure in Figure 2g).

**Figure 3:**
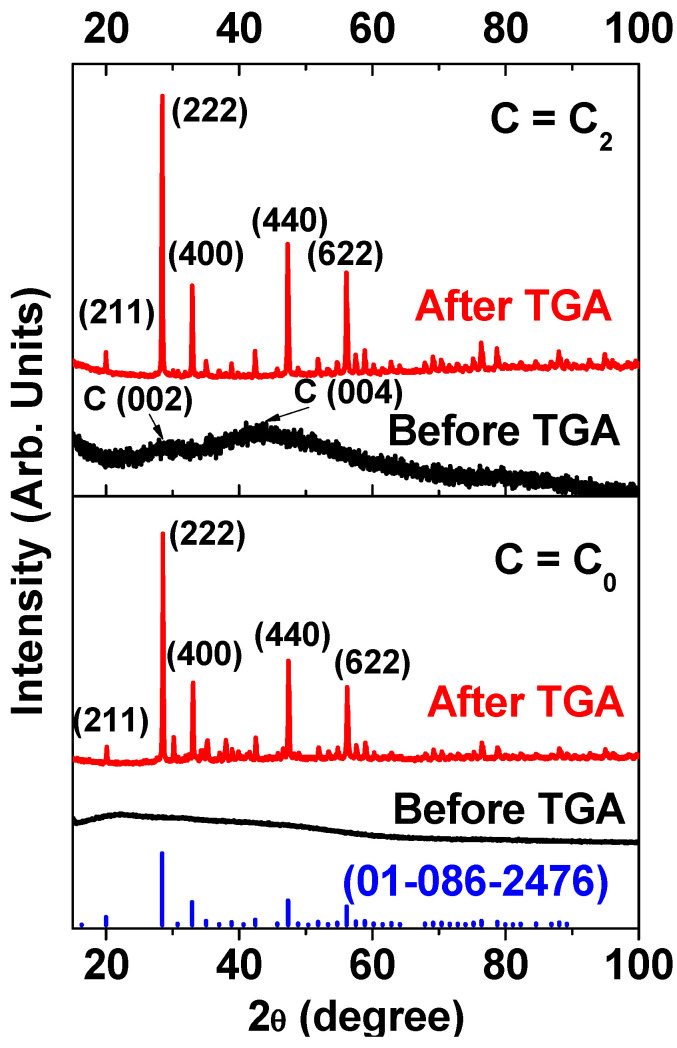
XRD patterns of powder sample of PDA-Eu_2_O_3_@C (C = C_0_ and C_2_) nanoparticles before and after TGA at temperatures up to 900 °C under airflow. The reference peaks of cubic Eu_2_O_3_ (JCPDS card No. 01-086-2476) are shown at the bottom [25].

The X-ray diffraction (XRD) patterns of powdered PDA-Eu_2_O_3_@C (C = C_0_ and C_2_) nanoparticles were measured before and after thermogravimetric analysis (TGA) (Figure 3). Before TGA for C = C_2_ sample, the nanoparticles showed broad peaks at 2θ values between ~29° and ~43°, corresponding to the C (002) and C (004) peaks of amorphous graphitic carbon, respectively, as observed in amorphous graphitic carbon materials [23,24], whereas such peaks were not observed for C = C_0_ sample. This result confirms the carbon grafting on the Eu_2_O_3_ nanoparticle surfaces. After TGA for C = C_2_ sample, the carbon peaks disappeared owing to the removal of the grafted carbon layers through combustion reaction under airflow. For both C = C_0_ and C_2_ samples, the sharp peaks corresponded to the cubic phase of Eu_2_O_3_, matching with JCPDS card No. 01-086-2476 [25]. The estimated lattice constant for the samples (10.851 Å) was consistent with the reported value of 10.868 Å [25].

#### 2.1.2. Quantification and Structure of Surface-Grafted Carbon Layers

**Figure 4:**
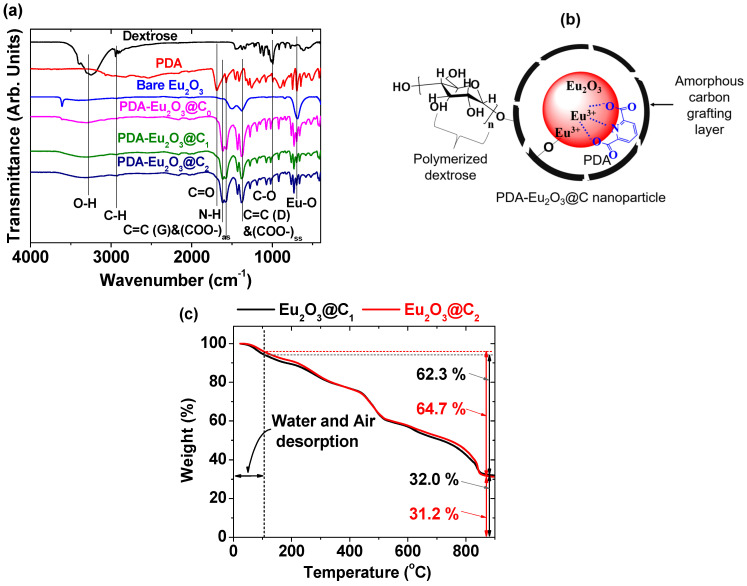
(**a**) FT-IR absorption spectra of PDA-Eu_2_O_3_@C nanoparticles (C = C_0_, C_1_, and C_2_), dextrose, PDA, and bare Eu_2_O_3_ nanoparticles; “as” and “ss” indicate asymmetric and symmetric stretching vibrations, respectively. (**b**) Proposed structure of grafted carbon layer and PDA conjugation on Eu_2_O_3_ nanoparticle surface. (**c**) TGA curves of Eu_2_O_3_@C nanoparticles (C = C_1_ and C_2_).

The surface grafting of Eu_2_O_3_ nanoparticles with carbon and PDA was investigated by recording Fourier transform infrared (FT-IR) absorption spectra of powdered PDA-Eu_2_O_3_@C nanoparticles (C = C_0_, C_1_, and C_2_) including dextrose and PDA as well as bare Eu_2_O_3_ nanoparticles as a reference (Figure 4a). Two specific peaks corresponding to the C=C stretching vibrations were observed at 1389 cm^−1^ (D-band) and 1573 cm^−1^ (G-band) [18,26,27] for the PDA-Eu_2_O_3_@C nanoparticles (C = C_1_ and C_2_) but not for dextrose, which contains no C=C bond; this confirms the successful amorphous graphitic carbon grafting on the Eu_2_O_3_ nanoparticle surfaces. The Eu–O stretching peak was observed at 696 cm^−1^ in the spectrum of the bare Eu_2_O_3_ nanoparticles, but its intensity decreased in the spectra of carbon- and PDA-grafted samples. The successful grafting of PDA on the nanoparticle surfaces, driven by strong Lewis acid–base interactions [28,29] between the Eu^3+^ (Lewis acids) on the nanoparticle surface and the two COO^−^ groups and one N group (Lewis bases) of each PDA, was confirmed by the splitting and redshift of the C=O symmetric stretching peak of PDA at 1688 cm^−1^ into 1389 (COO^−^ symmetric stretching) and 1573 cm^−1^ (COO^−^ antisymmetric stretching) peaks in the spectra of PDA-Eu_2_O_3_@C nanoparticles (C = C_0_, C_1_, and C_2_). These peaks overlapped with the G and D bands of the grafted carbon layers. The observed absorption frequencies are listed in Table 2. As shown in Figure 4b, the grafted carbon layer is made of almost randomly oriented aromatic carbon sheets and is terminated by polymerized dextrose, similar to the carbon nanoparticles [23,30]. The amorphous carbon can conjugate with Eu^3+^ on the Eu_2_O_3_ nanoparticle surface through an oxygen ion, and the numerous -OH groups of the polymerized dextrose on the outer carbon surfaces impart good colloidal stability to the Eu_2_O_3_@C nanoparticles in aqueous solution. The remaining free Eu^3+^ on the nanoparticle surface enables its conjugation with PDA.

The grafted carbon amounts on the Eu_2_O_3_@C nanoparticles (C = C_1_ and C_2_) were estimated to be 62.3 and 64.7 wt.%, respectively, based on the mass losses in the TGA curves (Figure 4c and Table 1), taking into account the initial mass losses of 5.7 and 4.1 wt% at temperatures up to ~105 °C, resulting from water and air desorption from the powder samples.

### 2.2. Ultraviolet (UV)–Visible Absorption and Photoluminescence Properties

#### 2.2.1. Determination of Optimal PDA Amount in PDA-Eu_2_O_3_@C Nanoparticles

**Figure 5:**
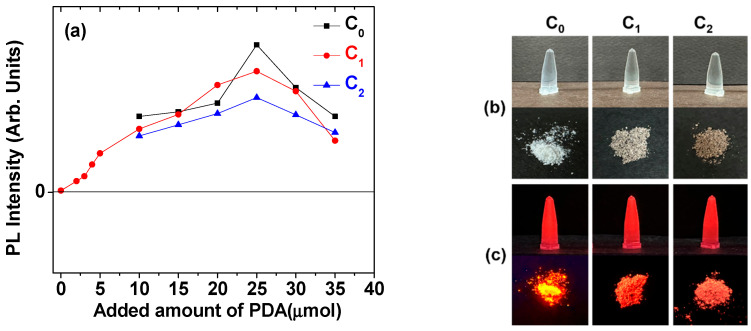
(**a**) Intensity plots of 615 nm peak of Eu^3+^ (^5^D_0_ → ^7^F_2_) in PDA-Eu_2_O_3_@C nanoparticles (C = C_0_, C_1_, and C_2_) dispersed in aqueous media and the solution sample photographs (C = C_1_) under 254 nm UV irradiation. Photographs of aqueous solution samples and powder PDA-Eu_2_O_3_@C nanoparticles (C = C_0_, C_1_, and C_2_) (**b**) without and (**c**) with 254 nm UV irradiation. 2 mM [Eu] for all solution samples.

To determine the optimal PDA amount in PDA-Eu_2_O_3_@C nanoparticles (C = C_0_, C_1_, and C_2_) to yield the maximum PL intensity, dilute sample solutions (2 mM [Eu]) were prepared, and PL spectra were recorded as a function of the added PDA amount in the synthesis, and the measured PL intensities of the 615 nm peak of Eu^3+^ (^5^D_0_ → ^7^F_2_) were plotted as a function of the added PDA amount (Figure 5a). As displayed in Figure 5a, the optimal PDA amount was determined to be ~25 µmol for all PDA-Eu_2_O_3_@C nanoparticle samples. As shown at the bottom figure in Figure 5a, the aqueous solution samples exhibited gradually increasing red color under 254 nm irradiation, with the brightest red color observed at ~25 µmol PDA; the aqueous solution samples of PDA-Eu_2_O_3_@C_1_ nanoparticles are presented as an example, and the other solution samples also showed similar color changes.

Figure 5b shows the above-prepared aqueous solution samples of PDA-Eu_2_O_3_@C nanoparticles (C = C_0_, C_1_, and C_2_) (2 mM [Eu]), along with corresponding powder samples obtained by drying the solution samples. In the absence of carbon grafting (C = C_0_), the solution sample was colorless, and the powder sample had a white appearance. However, the carbon grafted samples displayed a dark color and became darker as the grafted carbon amount increased from C_1_ to C_2_, confirming the carbon grafting. Under 254 nm UV irradiation, the aqueous solution and powder samples showed a red color due to Eu emission, whereas the non-grafted samples (i.e., C = C_0_) exhibited the strongest red color owing to no fluorescence quenching by carbon (Figure 5c).

#### 2.2.2. UV–Visible Absorption, Excitation, and PL Spectra

**Figure 6:**
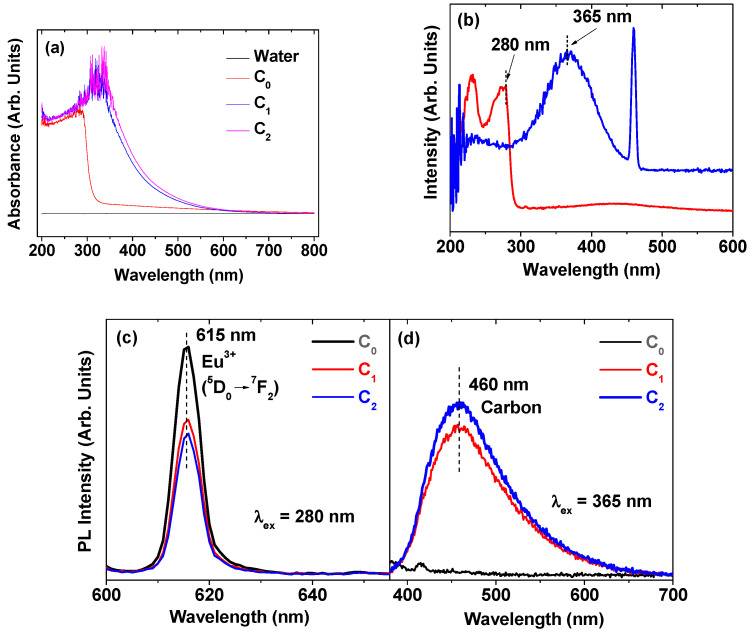
(**a**) UV–visible absorption spectra, (**b**) excitation spectra for 615 nm peak of Eu^3+^ (red) and 460 nm peak of grafted carbon layer (blue), and PL spectra of PDA–Eu_2_O_3_@C nanoparticles (C = C_0_, C_1_, and C_2_) dispersed in aqueous media, with λ_ex_ of (**c**) 280 and (**d**) 365 nm.

As observed in UV–visible absorption spectra (Figure 6a), the overall absorption intensity of the nanoparticle samples dispersed in aqueous media increased with the increase in the amount of grafted carbon from C_0_ to C_2_, confirming more absorptions by grafted carbon layers as they increased in number, which may reduce the PL intensity and QY of the nanoparticles.

To determine the excitation wavelengths (λ_ex_) of the samples, excitation spectra were recorded based on the strongest emission peak of Eu^3+^ at 615 nm [31,32,33,34,35,36,37,38] and the broad emission peak of amorphous carbon at 460 nm [18,39,40,41]. As displayed in Figure 6b, the λ_ex_ values for Eu^3+^ and the carbon-grafted layer were determined to be 280 and 365 nm, respectively.

The PL spectra of the PDA-Eu_2_O_3_@C nanoparticles (C = C_0_, C_1_, and C_2_) (λ_ex_ = 280 nm) were recorded to explore the carbon effects on the fluorescence intensities of Eu_2_O_3_ nanoparticles in aqueous media. As shown in Figure 6c, the PL intensity of the Eu^3+^ peak at 615 nm decreased with the increase in the amount of grafted carbon from C_0_ to C_2_, demonstrating the carbon fluorescent quenching effects on the PL intensity of Eu_2_O_3_ nanoparticles. On the other hand, the PL intensity of the grafted carbon layer peak at 460 nm (λ_ex_ = 365 nm) increased as the grafted carbon amount increased from C_0_ to C_2_, as shown in Figure 6d.

#### 2.2.3. Absolute QY and Fluorescence Lifetime

**Figure 7:**
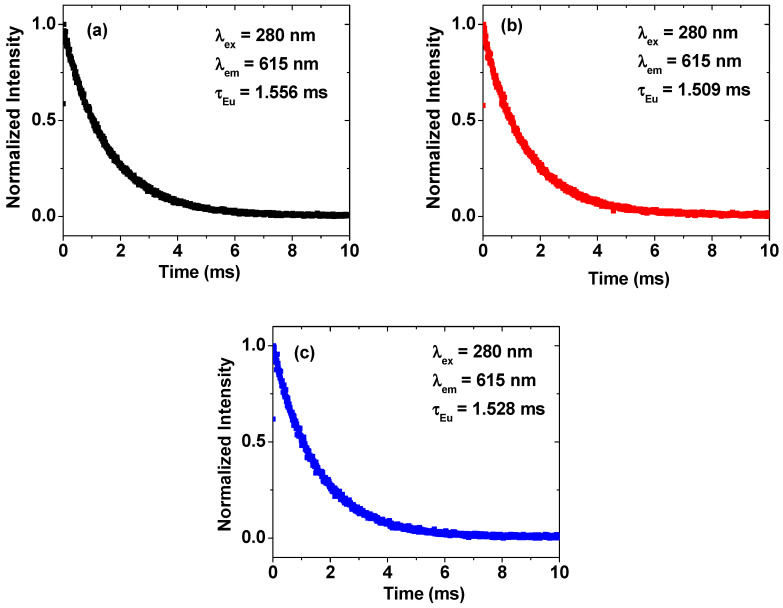
TRF spectra of PDA-Eu_2_O_3_@C nanoparticles [C = (**a**) C_0_, (**b**) C_1_, and (**c**) C_2_] dispersed in aqueous media. λ_ex_, λ_em_, and τ_Eu_ denote excitation wavelength, emission wavelength, and fluorescence lifetime, respectively.

The absolute QYs of PDA-Eu_2_O_3_@C nanoparticles (C = C_0_, C_1_, and C_2_) were measured using an integration sphere installed in a PL spectrometer (Table 3). As shown in Table 3, the absolute QY decreased with the increase in the amount of grafted carbon from C_0_ to C_2_, confirming the carbon fluorescent quenching effects on Eu_2_O_3_ nanoparticles. Fluorescence lifetimes (τ) were determined by measuring time-resolved fluorescence (TRF) spectra for the 615 nm peak of Eu^3+^ in PDA-Eu_2_O_3_@C nanoparticles (Figure 7). The τ values changed little with grafted carbon amount (Figure 7 and Table 3), indicating that the grafted carbon layer had a negligible effect on the fluorescence lifetime.

#### 2.2.4. Effect of Carbon Removal on PL Intensity

**Figure 8:**
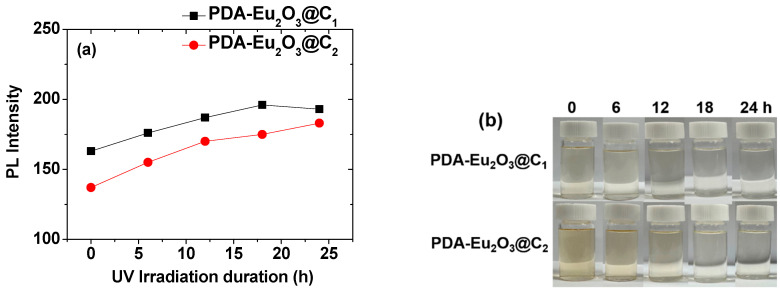
(**a**) Plots of PL intensity of 615 nm peak of Eu^3+^ in PDA-Eu_2_O_3_@C nanoparticles (C = C_1_ and C_2_) as a function of 365 nm UV irradiation time. (**b**) Aqueous solution sample photographs as a function of 365 nm UV irradiation time; the Eu concentration in the solution samples was 2 mM.

To further confirm the fluorescence quenching of the carbon layer, the removal effects of grafted carbon layer on the PL intensities of the PDA-Eu_2_O_3_@C nanoparticles were investigated. The carbon layer was removed through oxidation with H_2_O_2_ under 365 nm UV irradiation; this produced •OH radicals serving as oxidants to decompose the grafted carbon layer (indicated as C_n_H_m_O_k_), according to the following reaction [42,43,44]:

C_n_H_m_O_k_ + xH_2_O_2_ + UV → C_n_H_m_O_k_ + 2x•OH → yCO_2_ + zH_2_O

in which subscripts n, m, and k, and coefficients x, y, and z are arbitrary numbers. As shown in Figure 8a, the solution samples treated with H_2_O_2_ (0.1% in water) exhibited a gradually rising PL intensity with the increase in UV irradiation time owing to the increased removal of the grafted carbon layer. This result further confirms the fluorescence quenching effect of the carbon layer. As displayed in Figure 8b, the solution samples gradually changed from dark to colorless owing to the removal of the black carbon layer. The Eu_2_O_3_ nanoparticles have been used as doping materials to enhance the production of reactive radicals such as hydroxyl radicals to remove harmful organic molecules in aqueous media [45,46]. The Eu_2_O_3_ surface etching might occur during carbon layer removal owing to reactive hydroxyl radicals; however, this issue will require systematic investigation as a further study.

## 3. Discussion

In this study, carbon effects on fluorescence quenching of Eu_2_O_3_ nanoparticles were systematically investigated by constructing carbon shells of varying thicknesses to simulate carbon contaminations and investigate this issue. The carbon contaminations on nanoparticles may occur during synthesis when they are synthesized in organic solvents, along with or surface modification with organic molecules. In particular, the carbon contaminations will be severe at high temperature synthesis because organic molecules can be thermally decomposed at high temperatures. The carbon materials (i.e., carbon dots and carbon sheets) synthesized from various carbon precursors are mostly graphitic carbon materials with functional groups which are derived from carbon precursors [23,47]. The synthesized carbon shells using dextrose in this study are also graphitic carbon nanoparticles with many -OH groups derived from dextrose [18]. Therefore, the carbon layers in this study are similar to the common carbon contaminations generated during nanoparticle synthesis.

To investigate carbon effects on fluorescence quenching, PDA-Eu_2_O_3_@C nanoparticles (C = C_0_, C_1_, and C_2_) with different grafted amounts of carbon were synthesized by using different amounts of dextrose as a carbon source (C_0_ < C_1_ < C_2_; C_0_ = no carbon) to simulate carbon contaminations and investigate this issue. Then PL spectra and absolute QYs were measured. The PDA served as a photosensitizer to enhance PL intensity of Eu_2_O_3_ nanoparticles and the synthesized nanoparticles were stable as colloids in aqueous media.

The amorphous C (002) and C (004) carbon peaks in XRD patterns [23,24] (Figure 3) and the D-and G-bands at 1389 and 1573 cm^−1^, respectively, for the C=C bond vibrations in FT-IR absorption spectra [18,26,27] (Figure 4a) confirmed the presence of grafted carbon layers. In addition, the grafted carbon amounts were estimated to be 62.3 and 64.7 wt.% for C = C_1_ and C_2_, respectively, from TGA curves (Figure 4c). In addition, color of the aqueous solution samples and powdered samples became darker with the increase in the amount of grafted carbon from C_1_ to C_2_, with no color for C_0_ (Figure 2g and Figure 5b), confirming the grafted carbon layer on the nanoparticle surfaces. It is well-known that electronic states of 4f-electrons of Eu^3+^ are barely affected by chemical structure and bonding and composition. This is attributed to the compact 4f orbitals that are located close to the nucleus [48]. However, slight changes in 4f-electronic states may affect the luminescence properties (i.e., emission peak positions, emission intensity, and QY) of Eu_2_O_3_ cores. For example, variations in crystallinity can modify the local coordination symmetry, which may regulate the probability of f-f transitions, peak position, and peak intensity. In addition, amorphous samples typically can have a high density of defects, which may increase the probability of non-radiative transitions.

**Figure 9:**
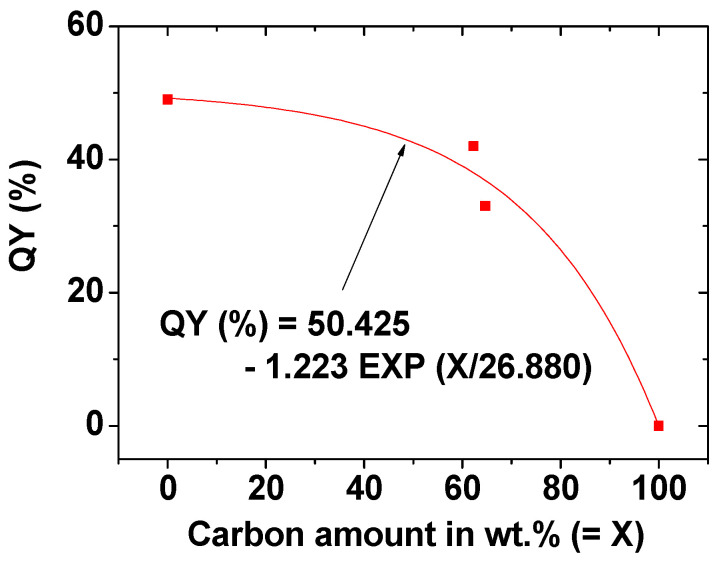
Plot of QY values as a function of grafted carbon amount in wt.% estimated from TGA (labeled as X), assuming an exponential decay and 0 value at X = 100%.

The previous studies investigated the fluorescence quenching of carbon nanoparticles as sensors by various materials such as metal ions, reactive oxygen species, metabolites, bacteria, viruses, and pathogens by detecting fluorescence intensity drop [49,50,51]. However, fluorescence quenching by carbon nanoparticles has not been reported. The fluorescence quenching effects of carbon-grafting layers were clearly demonstrated from PL intensity and QY drops (Figure 6c and Table 3) as the grafted carbon amount increased from C_0_ to C_2_. However, the PL intensity of the grafted carbon layers increased from C_0_ to C_2_ (Figure 6d) owing to the increased grafted amount of carbon. In particular, the fluorescence lifetimes (τ) of all nanoparticle samples were nearly the same at ~1.5 ms (Figure 7 and Table 3), indicating that the carbon grafting negligibly affects the fluorescence lifetime, ruling out dynamic quenching. This implies that the carbon grafting only reduces the PL intensity and QY by absorbing excitation light from light source and emission light from Eu_2_O_3_ nanoparticles; i.e., the carbon quenching is attributed to the inner filter effect [52,53] rather than molecular quenching because the amorphous carbon materials absorb UV–visible photons [18], as observed in the UV–visible absorption spectra (Figure 6a). Because the C_0_ sample does not contain carbon layers, the contribution of carbon layers to QYs by the inner filter effect was roughly estimated by using the QY difference: i.e., the inner filter effect in QY = QY (C_1_ or C_2_) − QY (C_0_). Therefore, the inner filter effect in QY for C_1_ sample is 49 − 42 = 7% and that for C_2_ sample is 49 − 33 = 16%. The fluorescence quenching effects of grafted carbon layers were further confirmed by removing them through oxidation with H_2_O_2_ under 365 nm UV irradiation and measuring PL intensity enhancement. The nanoparticles exhibited gradually increasing PL intensity with the increase in UV irradiation time owing to reduced grafted carbon amounts (Figure 8a). Assuming QY = 0 at 100 grafted carbon wt.% in nanoparticles and an exponential decay of QY, the QY decreases such as QY(%) = 50.425 − 1.223 EXP (X/26.880) (X = grafted carbon amount in wt.% estimated from TGA) (Figure 9). Therefore, it is better to avoid carbon contaminations in fluorescent nanoparticles during synthesis in order to achieve high QYs.

## 4. Materials and Methods

### 4.1. Chemicals

EuCl_3_·xH_2_O (99.99%), NaOH (99.99%), dextrose (C_6_H_12_O_6_) (>99.5%), dimethyl sulfoxide (DMSO, 99.9%), and trimethylammonium hydroxide pentahydrate (TMAH, ≥97%) were purchased from Sigma-Aldrich (St. Louis, MO, USA) and used as received. PDA (99%) was purchased from Tokyo Chemical Industry (TCI, Tokyo, Japan) and used as received. Ethanol (99%, Duksan, Ansan, Republic of Korea) was used for the initial washing of the nanoparticles. Triple-distilled water was used for the final washing of the nanoparticles and to prepare their suspension solutions.

### 4.2. Synthesis

To prepare Eu_2_O_3_ nanoparticles (Figure 1a), 2 mmol of EuCl_3_·xH_2_O precursor was dissolved in 20 mL of DMSO in a three-necked round-bottom flask suspended on a silicon oil bath on a hot plate, using magnetic stirring under atmospheric conditions. The subsequent addition of 2.6 mL of TMAH resulted in the appearance of a white cloudy mixture owing to nanoparticle formation. The mixture was magnetically stirred at 35 °C for 24 h and then washed three times with 400 mL of EtOH via centrifugation (4000 rpm). To remove any remaining impurities, the product solution was further dialyzed using a dialysis tube (MWCO = 2000 amu) against 1 L of triple-distilled water with magnetic stirring for 3 days; the triple-distilled water was replaced thrice during dialysis. To graft carbon on Eu_2_O_3_ nanoparticle surfaces (Figure 1b), a dextrose solution consisting of different amounts (i.e., 0, 4, and 8 mmol for C = C_0_, C_1_, C_2_, respectively) of dextrose in 10 mL of triple-distilled water was added to the synthesized Eu_2_O_3_ nanoparticle solution in a three-necked round-bottom flask. The mixed solution was magnetically stirred for 30 min. Then, a NaOH solution consisting of 4 mmol of NaOH in 10 mL of triple-distilled water placed in a 50 mL beaker was added to the above solution until its pH reached 10; the mixed solution was magnetically stirred at 95 °C until it became black. The obtained solution was cooled to room temperature, filtered with Whatman paper, transferred to a dialysis tube (MWCO = 2000 amu), and dialyzed against 1 L of triple-distilled water with magnetic stirring for 3 days to remove free dextrose and unreacted NaOH; triple-distilled water was replaced thrice during dialysis. To remove free carbon species from the obtained nanoparticles, the product solution was centrifuged at 4000 rpm for 60 min. Then, the supernatant top solution was removed, and the remaining product nanoparticles precipitated at the bottom of the centrifugation tube were redispersed in triple-distilled water: this centrifugation process was repeated thrice. To conjugate PDA with the Eu_2_O_3_ nanoparticles (Figure 1c), 4 mL of above Eu_2_O_3_@C nanoparticle solution (2 mM [Eu]) and different amounts of PDA (2–35 μmol) were mixed and simply shaken for 1 min to obtain PDA-Eu_2_O_3_@C nanoparticles. After the optimal PDA amount was determined from the highest PL intensity, the PDA conjugation reaction was repeated 10 times to increase the sample amount using the optimal amount of PDA. Then, the mixture was dialyzed against 1 L of triple distilled water (MWCO = 1000 amu) for 1 to remove free PDA. The obtained solution sample was divided into two equal volumes; one was dried to powder form for various characterizations, while the other was dispersed in triple-distilled water to prepare an aqueous nanoparticle suspension.

### 4.3. Characterizations

The diameters of the PDA-Eu_2_O_3_@C nanoparticles were measured using an HRTEM instrument (Titan G2 ChemiSTEM CS Probe, FEI, Hillsboro, OR, USA) operated at an accelerating voltage of 200 kV. For the measurements, one drop of diluted nanoparticle colloids in triple-distilled water was released onto a carbon film supported by a 200-mesh copper grid (PELCO no. 160, Ted Pella, Inc., Redding, CA, USA), which was placed on filter paper using a micropipette (2–20 mL, Eppendorf, Hamburg, Germany). The copper grid with the sample was allowed to dry in air at room temperature before being loaded into the HRTEM instrument for measurements. The hydrodynamic diameters (a) and zeta potentials (ζ) of PDA-Eu_2_O_3_@C nanoparticles in aqueous media were measured using diluted solution samples (0.1 mM [Eu]) and a particle size analyzer (Zetasizer Nano ZS, Malvern Panalytical, Malvern, UK).

The crystal structure of the powder samples before and after TGA was measured using a multi-purpose powder XRD spectrometer (X-PERT PRO MRD, Philips, Eindhoven, The Netherlands) with unfiltered CuKa (*λ* = 1.54184 Å) radiation. The scanning step and scan range in 2*θ* were 0.03° and 15–100°, respectively.

The carbon attachment on PDA-Eu_2_O_3_@C nanoparticles was investigated by recording FT-IR absorption spectra (Galaxy 7020A, Mattson Instruments, Inc., Madison, WI, USA). For the measurements, the powder samples were placed on a hot plate at ~40 °C for 1 week to remove moisture. Then, dried powder samples were prepared as KBr pellets. FT-IR absorption spectra were recorded in the range of 400–4000 cm^−1^.

The grafted carbon amounts were estimated from TGA curves (SDT-Q600, TA Instruments, New Castle, DE, USA) of Eu_2_O_3_@C nanoparticles between room temperature and 900 °C under airflow. In particular, the grafted carbon amounts were estimated from the weight losses in the TGA curves after subtracting the initial weight losses between room temperature and ~105 °C, due to water and air desorption.

The Eu concentration in PDA-Eu_2_O_3_@C nanoparticles suspended in triple-distilled water was measured using inductively coupled plasma-atomic emission spectrometry (ICP-AES; Optima 7300DV, Perkin Elmer, Waltham, MA, USA). The colloidal suspension was subjected to acid pretreatment to completely dissolve the nanoparticles in solution prior to measurements.

A UV–visible absorption spectrometer (Shimadzu, UV-3600i plus, Tokyo, Japan) was used to measure UV–visible absorption spectra using aqueous nanoparticle suspension samples (0.1 mM [Eu]). A quartz cuvette with two optically clear sides was filled with a nanoparticle suspension sample to record UV–visible absorption spectra. A PL spectrometer (Cary Eclipse, Agilent Technologies, Santa Clara, CA, USA) was used to measure PL and TRF spectra using aqueous nanoparticle suspension samples. A quartz cuvette with four optically clear sides was filled with a nanoparticle suspension sample to record PL and TRF spectra. The sample concentration and slit width were 2 mM [Eu] and 5 nm, respectively. The absolute QYs were measured using an integration sphere installed in a PL spectrometer (Hitachi, F-7000, Tokyo, Japan). To determine absolute QYs, aqueous nanoparticle samples were filled into 0.2 mL polypropylene tubes, and their PL spectra were measured. Reference PL spectra were recorded using an empty polypropylene tube. In addition, the absolute QY of rhodamine 123 in ethanol (λ_ex_ = 500 nm) was measured to be 90.5%, in agreement with the reported value of 90%.

## 5. Conclusions

In summary, the fluorescent quenching effect of carbon, which has not previously investigated, was systematically investigated by synthesizing PDA-Eu_2_O_3_@C core–shell nanoparticles (core d_avg_ = 2.4 nm) with different amounts of grafted carbon layers on Eu_2_O_3_ nanoparticles to simulate carbon contaminations and investigate this issue, followed by measuring PL spectra and absolute QYs. We found that as the grafted carbon amount increased, the PL intensity and QY of Eu_2_O_3_ nanoparticles decreased, demonstrating the fluorescent quenching effect of carbon. In addition, the oxidation removal of grafted carbon layers from PDA-Eu_2_O_3_@C nanoparticles using H_2_O_2_ and 365 nm UV irradiation resulted in enhanced PL intensity as the UV irradiation time (or carbon removal amount) increased, further confirming the fluorescence quenching effect of carbon. Therefore, the present results indicate that it is better to avoid carbon contaminations in fluorescent nanoparticles during synthesis in order to achieve high QYs.

## Figures and Tables

**Table 1:** Summarized properties of PDA-Eu_2_O_3_@C nanoparticles (C = C_0_, C_1_, and C_2_).

Sample	d_avg_ (nm)	a_avg_ (nm)	ξ_avg_ (mV)	Surface-Grafting Result	
				Grafted Carbon Amount (wt.%)	Nanoparticle Amount (wt.%)
C_0_	2.4	-	-	-	-
C_1_	2.4	32.9	−29.7	62.3	32.0
C_2_	2.4	34.1	−20.4	64.7	31.2

**Table 2:** Observed FT-IR absorption frequencies (cm^−1^).

Sample	O–H	C–H	C=O	N–H	C=C (G)	C=C (D)	(COO^−^)_as_	(COO^−^)_ss_	C–O	Eu–O
Dextrose	3242	2940	-		-	-	-	-	990	-
PDA	-	-	1688		-	-	-	-	-	-
Bare Eu_2_O_3_	-	-	-	-	-	-	-	-	-	696
PDA-Eu_2_O_3_@C_0_	3242	-	-	1612	-	-	1573	1389	-	~710
PDA-Eu_2_O_3_@C_1_	3242	2940	-	1612	1573	1389	1573	1389	-	~710
PDA-Eu_2_O_3_@C_2_	3242	2940	-	1612	1573	1389	1573	1389	-	~710

**Table 3:** Absolute QY and τ values of PDA-Eu_2_O_3_@C nanoparticles (C = C_0_, C_1_, and C_2_) dispersed in aqueous media.

Sample	QY (%)	τ (ms)
C_0_	49	1.556
C_1_	42	1.509
C_2_	33	1.528

## Data Availability

The original contributions presented in this study are included in the article. Further inquiries can be directed to the corresponding authors.

## References

[1] Hong G., Antaris A.L., Dai H. (2017). Near-infrared fluorophores for biomedical imaging. Nat. Biomed. Eng..

[2] Rao J., Dragulescu-Andrasi A., Yao H. (2007). Fluorescence imaging in vivo: Recent advances. Curr. Opin. Biotechnol..

[3] Heilemann M., van de Linde S., Schüttpelz M., Kasper R., Seefeldt B., Mukherjee A., Tinnefeld P., Sauer M. (2008). Subdiffraction-resolution fluorescence imaging with conventional fluorescent probes. Angew. Chem. Int. Ed..

[4] Alivisatos P. (2004). The use of nanocrystals in biological detection. Nat. Biotechnol..

[5] Xiao D., Qi H., Teng Y., Pierre D., Kutoka P.T., Liu D. (2021). Advances and challenges of fluorescent nanomaterials for synthesis and biomedical applications. Nanoscale Res. Lett..

[6] Link S., El-Sayed M.A. (2003). Optical properties and ultrafast dynamics of metallic nanocrystals. Ann. Rev. Phys. Chem..

[7] Chopada R., Sarwata R., Kumar V. (2025). Effect of mild to extreme pH, temperature, and ionic strength on the colloidal stability of differentially capped gold nanoparticles. J. Mol. Struct..

[8] Drozdick H.K., Weiss R., Sullivan C.M., Wieghold S., Nienhaus L. (2022). Widespread opportunities for materials engineering of nanocrystals: Synthetically tailorable effects and methodologies. Matter.

[9] Yi Z., Luo Z., Qin X., Chen Q., Liu X. (2020). Lanthanide-Activated Nanoparticles: A Toolbox for Bioimaging, Therapeutics, and Neuromodulation. Acc. Chem. Res..

[10] Bünzli J.C.G. (2010). Lanthanide luminescence for biomedical analyses and imaging. Chem. Rev..

[11] Zhang Q., O’Brien S., Grimm J. (2022). Biomedical Applications of Lanthanide Nanomaterials, for Imaging, Sensing and Therapy. Nanotheranostics.

[12] Dong H., Du S.-R., Zheng X.-Y., Lyu G.-M., Sun L.-D., Li L.-D., Zhang P.-Z., Zhang C., Yan C.-H. (2015). Lanthanide Nanoparticles: From Design toward Bioimaging and Therapy. Chem. Rev..

[13] Thomas S.W., Joly G.D., Swager T.M. (2007). Chemical sensors based on amplifying fluorescent conjugated polymers. Chem. Rev..

[14] Fan C., Wang S., Hong J.W., Bazan G.C., Plaxco K.W., Heeger A.J. (2003). Beyond superquenching: Hyper-efficient energy transfer from conjugated polymers to gold nanoparticles. Proc. Natl. Acad. Sci. USA.

[15] Wu C., Zheng Y., Szymanski C., McNeill J. (2008). Energy transfer in a nanoscale multichromophoric system: Fluorescent dye-doped conjugated polymer nanoparticles. J. Phys. Chem. C.

[16] van de Weert M. (2010). Fluorescence quenching to study protein-ligand binding: Common errors. J. Fluoresc..

[17] Lyu D., Li J., Wang X., Guo W., Wang E. (2018). Cationic-polyelectrolyte-modified fluorescent DNA–silver nanoclusters with enhanced emission and higher stability for rapid bioimaging. Anal. Chem..

[18] Tegafaw T., Oh I.T., Cha H., Yue H., Miao X., Ho S.L., Ahmad M.Y., Marasini S., Ghazanfari A., Kim H.K. (2018). Facile synthesis of stable colloidal suspension of amorphous carbon nanoparticles in aqueous medium and their characterization. J. Phys. Chem. Solids.

[19] Bhunia S.K., Saha A., Maity A.R., Ray S.C., Jana N.R. (2013). Carbon nanoparticle-based fluorescent bioimaging probes. Sci. Rep..

[20] Lim S.Y., Shen W., Gao Z. (2015). Carbon quantum dots and their applications. Chem. Soc. Rev..

[21] Yue H., Marasini S., Ahmad M.Y., Ho S.L., Cha H., Liu S., Jang Y.J., Tegafaw T., Ghazanfari A., Miao X. (2020). Carbon-coated ultrasmall gadolinium oxide (Gd_2_O_3_@C) nanoparticles: Application to magnetic resonance imaging and fluorescence properties. Colloids Surf. A Physicochem. Eng. Asp..

[22] Yue H., Park J.A., Ho S.L., Ahmad M.Y., Cha H., Liu S., Tegafaw T., Marasini S., Ghazanfari A., Kim S. (2020). New Class of Efficient T_2_ Magnetic Resonance Imaging Contrast Agent: Carbon-Coated Paramagnetic Dysprosium Oxide Nanoparticles. Pharmaceuticals.

[23] Hara M., Yoshida T., Takagaki A., Takata T., Kondo J.N., Hayashi S., Domen K. (2004). A carbon material as a strong protonic acid. Angew. Chem. Int. Ed..

[24] Tsubouchi N., Xu C., Ohtsuka Y. (2003). Carbon crystallization during high-temperature pyrolysis of coals and the enhancement by calcium. Energy Fuels.

[25] Morris M., McMurdie H., Evans E., Paretzkin B., Parker H., Pyrros N., Hubbard C. (1984). Standard X-Ray Diffraction Powder Patterns: Section 20—Data for 71 Substances.

[26] Kaufman J.H., Metin S. (1989). Symmetry breaking in nitrogen-doped amorphous carbon: Infrared observation of the Raman-active G and D bands. Phys. Rev. B.

[27] Malard L.M., Pimenta M.A., Dresselhaus G., Dresselhaus M.S. (2009). Raman spectroscopy in graphene. Phys. Rep..

[28] Pearson R.G. (1968). Hard and soft acids and bases, HSAB, part I: Fundamental principles. J. Chem. Educ..

[29] Pearson R.G. (1968). Hard and soft acids and bases, HSAB, part II: Underlying theories. J. Chem. Educ..

[30] Sun X., Li Y. (2004). Colloidal carbon spheres and their core/shell structures with noble metal nanoparticles. Angew. Chem. Int. Ed..

[31] Tegafaw T., Liu Y., Ho S.L., Liu S., Ahmad M.Y., Al Saidi A.K.A., Zhao D., Ahn D., Nam H., Chae W.S. (2023). High-Quantum-Yield Ultrasmall Ln_2_O_3_ (Ln = Eu, Tb, or Dy) Nanoparticle Colloids in Aqueous Media Obtained via Photosensitization. Langmuir.

[32] Petoud S., Cohen S.M., Bünzli J.C.G., Raymond K.N. (2003). Stable lanthanide luminescence agents highly emissive in aqueous solution: Multidentate 2-hydroxyisophthalamide complexes of Sm^3+^, Eu^3+^, Tb^3+^, Dy^3+^. J. Am. Chem. Soc..

[33] Mohamed H.E.A., Hkiri K., Khenfouch M., Dhlamini S., Henini M., Maaza M. (2020). Optical properties of biosynthesized nanoscaled Eu_2_O_3_ for red luminescence applications. J. Opt. Soc. Am. A.

[34] Mothudi B.M., Ntwaeaborwa O.M., Botha J.R., Swart H.C. (2009). Photoluminescence and phosphorescence properties of MAl_2_O_4_:Eu^2+^, Dy^3+^ (M=Ca, Ba, Sr) phosphors prepared at an initiating combustion temperature of 500 °C). Phys. B Condens. Matter..

[35] Tatte S.P., Parauha Y.R., Dhoble N.S., Ghanty C., Dhoble S.J. (2021). Combustion synthesis and spectroscopic properties of Ba_2_Zn_7_F_18_:Eu^3+^ phosphor. J. Phys. Conf. Ser..

[36] Yan T., Zhang D., Shi L., Li H. (2009). Facile synthesis, characterization, formation mechanism and photoluminescence property of Eu_2_O_3_ nanorods. J. Alloys Comp..

[37] Diallo A., Mothudi B.M., Manikandan E., Maaza M. (2016). Luminescent Eu_2_O_3_ nanocrystals by *Aspalathus linearis*’ extract: Structural and optical properties. J. Nanophotonics.

[38] Gedanken A., Reisfeld R., Sominski L., Zhong Z., Koltypin Y., Panczer G., Gaft M., Minti H. (2000). Time-dependence of luminescence of nanoparticles of Eu_2_O_3_ and Tb_2_O_3_ deposited on and doped in alumina. Appl. Phys. Lett..

[39] Zhang P., Zheng Y., Ren L., Li S., Feng M., Zhang Q., Qi R., Qin Z., Zhang J., Jiang L. (2024). The Enhanced Photoluminescence Properties of Carbon Dots Derived from Glucose: The Effect of Natural Oxidation. Nanomaterials.

[40] Stan C.S., Elouakassi N., Albu C., Ania C.O., Coroaba A., Ursu L.E., Popa M., Kaddami H., Almaggoussi A. (2024). Photoluminescence of Argan-Waste-Derived Carbon Nanodots Embedded in Polymer Matrices. Nanomaterials.

[41] Newman Monday Y., Abdullah J., Yusof N.A., Abdul Rashid S., Shueb R.H. (2021). Facile Hydrothermal and Solvothermal Synthesis and Characterization of Nitrogen-Doped Carbon Dots from Palm Kernel Shell Precursor. Appl. Sci..

[42] Glaze W.H., Beltran F., Tuhkanen T., Kang J.W. (1992). Chemical models of advanced oxidation processes. Water Qual. Res. J..

[43] Shu H.Y., Hsieh W.P. (2006). Treatment of dye manufacturing plant effluent using an annular UV/H_2_O_2_ reactor with multi-UV lamps. Sep. Purif. Technol..

[44] Saritha P., Aparna C., Himabindu V., Anjaneyulu Y. (2007). Comparison of various advanced oxidation processes for the degradation of 4-chloro-2 nitrophenol. J. Hazard. Mater..

[45] Liu J., Li Z., Wang M., Jin C., Kang J., Tang Y., Li S. (2021). Eu_2_O_3_/Co_3_O_4_ nanosheets for levofloxacin removal via peroxymonosulfate activation: Performance, mechanism and degradation pathway. Sep. Purif. Technol..

[46] Piao M., Liu X., Du H., Zhao L., Teng H. (2024). Preparation of Eu_2_O_3_/BiOBr for efficient photocatalytic thiamethoxam from aqueous solution. Desalin. Water Treat..

[47] Liu J., Li R., Yang B. (2020). Carbon Dots: A New Type of Carbon-Based Nanomaterial with Wide Applications. ACS Cent. Sci..

[48] Cotton F.A., Wilkinson G. (1980). Advanced Inorganic Chemistry.

[49] Krasley A.T., Li E., Galeana J.M., Bulumulla C., Beyene A.G., Demirer G.S. (2024). Carbon Nanomaterial Fluorescent Probes and Their Biological Applications. Chem. Rev..

[50] Lou X.-T., Zhan L., Chen B.-B. (2025). Recent Progress of Carbon Dots in Fluorescence Sensing. Inorganics.

[51] Han G., Cai J., Yang L., Li X., Wang X. (2024). Fluorescent Paper Based on CQDs/Rhodamine B: A Ratio and Sensitive Detection Platform for On-Site Fe^3+^ Sensing. Molecules.

[52] Weitner T., Friganović T., Šakić D. (2022). Inner Filter Effect Correction for Fluorescence Measurements in Microplates Using Variable Vertical Axis Focus. Anal. Chem..

[53] Kubista M., Sjöback R., Eriksson S., Albinsson B. (1994). Experimental Correction for the Inner-filter Effect in Fluorescence Spectra. Analyst.

